# Rapid typing of *Klebsiella pneumoniae* and *Pseudomonas aeruginosa* by Fourier-transform Infrared spectroscopy informs infection control in veterinary settings

**DOI:** 10.3389/fmicb.2024.1334268

**Published:** 2024-02-02

**Authors:** Flavia Zendri, Vanessa Schmidt, Norman Mauder, Anette Loeffler, Rosanne Ellen Jepson, Cajsa Isgren, Gina Pinchbeck, Sam Haldenby, Dorina Timofte

**Affiliations:** ^1^Department of Veterinary Anatomy, Physiology and Pathology, Institute of Infection, Veterinary and Ecological Sciences, University of Liverpool, Neston, United Kingdom; ^2^Department of Small Animal Clinical Science, Institute of Infection, Veterinary and Ecological Sciences, University of Liverpool, Neston, United Kingdom; ^3^Bruker Daltonics, Bremen, Germany; ^4^Department of Clinical Science and Services, Royal Veterinary College Hawkshead Campus, Hatfield, Hertfordshire, United Kingdom; ^5^Western Counties Equine Hospital Ltd., Culmstock, United Kingdom; ^6^Department of Livestock and One Health, Institute of Infection, Veterinary and Ecological Sciences, University of Liverpool, Neston, United Kingdom; ^7^Centre for Genomic Research, University of Liverpool, Liverpool, United Kingdom

**Keywords:** veterinary, infection control, Fourier-transform infrared (FTIR) spectroscopy, veterinary settings, companion animals, *Klebsiella pneumoniae*, *Pseudomonas aeruginosa*

## Abstract

**Introduction:**

The emergence of multi-drug resistant (MDR) pathogens linked to healthcare-associated infections (HCAIs) is an increasing concern in modern veterinary practice. Thus, rapid bacterial typing for real-time tracking of MDR hospital dissemination is still much needed to inform best infection control practices in a clinically relevant timeframe. To this end, the IR Biotyper using Fourier-Transform InfraRed (FTIR) spectroscopy has the potential to provide fast cluster analysis of potentially related organisms with substantial cost and turnaround time benefits.

**Materials and methods:**

A collection of MDR bacterial isolates (*n* = 199, comprising 92 *Klebsiella pneumoniae* and 107 *Pseudomonas aeruginosa*) obtained from companion animal (i.e., dogs, cats and horses) clinical investigations, faecal and environmental screening from four veterinary facilities between 2012 and 2019 was analysed retrospectively by FTIR spectroscopy. Its performance was compared against MLST extracted from whole genomes of a subset of clustering isolates (proportionally to cluster size) for investigation of potential nosocomial transmission between patients and the surrounding hospital environments.

**Results:**

Concordance between the FTIR and MLST types was overall high for *K. pneumoniae* (Adjusted Rand Index [ARI] of 0.958) and poor for *P. aeruginosa* (ARI of 0.313). FTIR *K. pneumoniae* clusters (*n* = 7) accurately segregated into their respective veterinary facility with evidence of intra-hospital spread of *K. pneumoniae* between patients and environmental surfaces. Notably, *K. pneumoniae* ST147 intensely circulated at one Small Animal Hospital ICU. Conversely, *Pseudomonas aeruginosa* FTIR clusters (*n* = 18) commonly contained isolates of diversified hospital source and heterogeneous genetic background (as also genetically related isolates spread across different clusters); nonetheless, dissemination of some clones, such as *P. aeruginosa* ST2644 in the equine hospital, was apparent. Importantly, FTIR clustering of clinical, colonisation and/or environmental isolates sharing genomically similar backgrounds was seen for both MDR organisms, highlighting likely cross-contamination events that led to clonal dissemination within settings.

**Conclusion:**

FTIR spectroscopy has high discriminatory power for hospital epidemiological surveillance of veterinary *K. pneumoniae* and could provide sufficient information to support early detection of clonal dissemination, facilitating implementation of appropriate infection control measures. Further work and careful optimisation need to be carried out to improve its performance for typing of *P. aeruginosa* veterinary isolates.

## 1 Introduction

Healthcare-associated infections (HCAIs) and the emergence of multi-drug resistant (MDR) nosocomial pathogens in companion animal (dogs, cats and horses) medicine have been increasingly reported in the last two decades, where outbreaks are often associated with, and complicated by, the antimicrobial resistant and zoonotic nature of the pathogens involved (Murphy et al., 2010; Steneroden et al., 2010; Wieler et al., 2011; Walther et al., 2014, 2018; Soza-Ossandón et al., 2020). Advancements in modern veterinary practice, including the progress in patient management, medical procedures and the development of hospital facilities, have given rise to favourable conditions for harbouring veterinary nosocomial MDR opportunistic pathogens, as seen in human hospitals. Despite this, the frequency and nature of HCAIs in veterinary hospitals are not known and the progress made in the field of veterinary infection control (IC) has been slow compared to human medicine (Weese, 2011; Walther et al., 2017). Nowadays, IC is an essential component in the operation of all veterinary hospitals delivering high-quality care; risks associated with lack of biosecurity and infection control programs include outbreaks of HCAIs in hospitalised patients and zoonotic infections of hospital personnel and animal owners, leading to increased morbidity, mortality, medical costs and welfare issues in both animals and people. Screening hospital surfaces in conjunction with cases of clinical infection is advisable as contaminated hospital environments may be an important source of infections in hospitalised human (Weber et al., 2013; Nutman et al., 2016) and animal patients (Weese et al., 2004; Loeffler et al., 2005; Hoet et al., 2011). Furthermore, MDR pathogens can persist in the hospital environment for long periods of time, providing continuous exposure for colonisation and subsequent infection in hospitalised patients (Kramer et al., 2006; Bortolami et al., 2017; Keck et al., 2020).

Rapid typing of bacterial isolates is valuable for outbreak management and hospital surveillance of MDR pathogens by revealing their clonal relationships and possible routes of transmission and by linking clinical isolates to environmental reservoirs (Foxman et al., 2005). These results can guide the implementation of focused interventions to tackle and prevent HCAIs. However, the quick detection of pathogen cross-transmissions in healthcare settings remains challenging despite the broad choice of typing methods available. Molecular typing methods relying on genomic fingerprinting, such as Pulsed-Field Gel Electrophoresis (PFGE), Multi-Locus Sequence Typing (MLST) and Whole Genome Sequencing (WGS) are widely used in hospital epidemiological surveillance and outbreak investigation to discern and track patterns of infection and transmission sources (Boccia et al., 2015; Timofte et al., 2016). However, while molecular tools are the gold standard for diagnostic accuracy and discriminatory power, they are mainly used in retrospective epidemiological studies, thereby lacking clinical applicability. Hence, a quick and reliable typing method detecting pathogen cross-transmissions in the clinical microbiology laboratory is still needed.

Fourier-Transform InfraRed (FTIR) spectroscopy is a phenotypic method generating highly-specific metabolic fingerprint-like signatures (from nucleic acids, proteins, carbohydrates and lipids) that are widely used to differentiate, identify and classify a range of microbial species and strains (Yang et al., 2020). The principle of this technique is that the absorption of the infrared (IR) radiation by bacterial cells causes excitation of the different molecules; different cell components absorb radiation at different wavelengths, originating characteristic spectral peaks that present as a specific fingerprint-like pattern (Novais et al., 2019). The potential of this technique as a quick (results can be available as early as 2–3 h from culture harvest), inexpensive, and high-throughput tool for bacterial typing is widely accepted, which makes it an attractive alternative to current gold standard typing methods for routine diagnostic applications (Finlayson et al., 2019).

Clinical research exploring the performance of FTIR in human hospitals indicated this could be a promising tool for infection control purposes, particularly for fast typing of gram-negative organisms associated with HCAIs’ outbreaks (Dinkelacker et al., 2018; Martak et al., 2019; Vogt et al., 2019; Rakovitsky et al., 2020). In our previous work exploring rapid bacterial typing of veterinary hospital-associated MDR bacteria, we reported unexpectedly high circulation of MDR gram-negative organisms belonging to the ESKAPE group of pathogens (*Enterococcus faecium, Staphylococcus aureus, Klebsiella pneumoniae, Acinetobacter baumannii, Pseudomonas aeruginosa* and *Enterobacter* spp.) within the intensive care units (ICUs) of two veterinary hospitals (one equine and one small animal; Zendri et al., 2023). Here, we consider the integration of this technology into infection control programmes by assessing its value in strain typing of *Klebsiella pneumoniae* and *Pseudomonas aeruginosa* from clinical cases, colonised inpatients and clinical environments in veterinary clinics/hospitals.

## 2 Materials and methods

### 2.1 Bacterial isolates

Sixty-seven isolates were selected from a previous pilot study (PS) which evaluated the epidemiology of multidrug resistant gram-negative (MDR-GN) bacteria in the ICUs of two veterinary hospitals, one small animal (SAH1) and one equine (EH), at the University of Liverpool (UoL), United Kingdom (Zendri et al., 2023), where *n* = 31 *K. pneumoniae* (Table 1) and *n* = 36 *P. aeruginosa* (Table 2) isolated across both hospitals, were selected for FTIR testing.

**Table 1:** Sample type and origin of clinical, faecal and environmental MDR *Klebsiella pneumoniae* spp. *pneumoniae* (*n* = 92) from four companion animal veterinary facilities in the UK (2012–2019).

Isolate ID	ICU PS	Hospital	Date collected	Sample type	Animal species	Site	Isolate
K1	Yes	SAH1	09/05/2018	Faecal	Dog	ICU	*Klebsiella pneumoniae*
K2		SAH1	09/05/2018	Environmental	–	ICU Kennel	*Klebsiella pneumoniae*
K3		SAH1	09/05/2018	Environmental	–	ICU Door Plate	*Klebsiella pneumoniae*
K4		SAH1	09/05/2018	Environmental	–	ICU Telephone	*Klebsiella pneumoniae*
K5		SAH1	09/05/2018	Environmental	–	ICU Floor	*Klebsiella pneumoniae*
K6		SAH1	11/05/2018	Environmental	–	ICU Kennel	*Klebsiella pneumoniae*
K7		SAH1	11/05/2018	Environmental	–	ICU Floor	*Klebsiella pneumoniae*
K8		SAH1	11/05/2018	Environmental	–	ICU Door Plate	*Klebsiella pneumoniae*
K9		SAH1	11/05/2018	Environmental	–	ICU Keyboard	*Klebsiella pneumoniae*
K10		SAH1	16/05/2018	Clinical	Dog	ICU Abdominal Fluid	*Klebsiella pneumoniae*
K11		SAH1	16/05/2018	Environmental	–	ICU Keyboard	*Klebsiella pneumoniae*
K12		SAH1	11/05/2018	Faecal	Dog	ICU	*Klebsiella pneumoniae*
K13		SAH1	18/05/2018	Faecal	Dog	ICU	*Klebsiella pneumoniae*
K14		SAH1	18/05/2018	Environmental	–	ICU Kennel	*Klebsiella pneumoniae*
K15		SAH1	18/05/2018	Environmental	–	ICU Floor	*Klebsiella pneumoniae*
K16		SAH1	18/05/2018	Environmental	–	ICU Keyboard	*Klebsiella pneumoniae*
K17		SAH1	18/05/2018	Environmental	–	ICU Telephone	*Klebsiella pneumoniae*
K18		SAH1	18/05/2018	Environmental	–	ICU Door Plate	*Klebsiella pneumoniae*
K19		SAH1	18/05/2018	Environmental	–	ICU Kennel	*Klebsiella pneumoniae*
K20		SAH1	18/05/2018	Environmental	–	ICU Floor	*Klebsiella pneumoniae*
K21		SAH1	18/05/2018	Environmental	–	ICU Telephone	*Klebsiella pneumoniae*
K22		SAH1	18/05/2018	Environmental	–	ICU Keyboard	*Klebsiella pneumoniae*
K23		SAH1	18/05/2018	Environmental	–	ICU Door Plate	*Klebsiella pneumoniae*
K24		SAH1	22/05/2018	Environmental	–	ICU Keyboard	*Klebsiella pneumoniae*
K25		SAH1	22/05/2018	Environmental	–	ICU Kennel	*Klebsiella pneumoniae*
K26		SAH1	22/05/2018	Environmental	–	ICU Door Plate	*Klebsiella pneumoniae*
K27		SAH1	22/05/2018	Environmental	–	ICU Floor	*Klebsiella pneumoniae*
K28		SAH1	22/05/2018	Environmental	–	ICU Telephone	*Klebsiella pneumoniae*
K29		SAH1	22/05/2018	Faecal	Dog	ICU	*Klebsiella pneumoniae*
K30		SAH1	25/05/2018	Faecal	Dog	ICU	*Klebsiella pneumoniae*
K31		SAH1	25/05/2018	Environmental	–	ICU Floor	*Klebsiella pneumoniae*
K32	No	SAH1	09/05/2018	Environmental	–	ICU Door Handle	*Klebsiella pneumoniae*
K33		SAH1	14/08/2017	Environmental	–	Laboratory Fridge	*Klebsiella pneumoniae*
K34		EH	23/06/2016	Environmental	–	Stable A10	*Klebsiella pneumoniae*
K35		SAH1	01/08/2016	Environmental	–	ICU Door Handle	*Klebsiella pneumoniae*
K36		SAH1	18/07/2016	Environmental	–	ICU Keyboard	*Klebsiella pneumoniae*
K37		SAH1	18/07/2016	Environmental	–	Student Locker	*Klebsiella pneumoniae*
K38		SAH1	12/07/2016	Environmental	–	Reception Photocopier	*Klebsiella pneumoniae*
K39		SAH1	09/03/2018	Clinical	Dog	Urine	*Klebsiella pneumoniae*
K40		SAH1	17/01/2018	Clinical	Dog	Faeces	*Klebsiella pneumoniae*
K41		EH	28/06/2013	Environmental	–	Stable A6	*Klebsiella pneumoniae*
K42		EH	17/03/2016	Environmental	–	Student Keyboard	*Klebsiella pneumoniae*
K43		SAH1	01/12/2017	Environmental	–	Dermatology Keyboard	*Klebsiella pneumoniae*
K44		SAH1	07/01/2016	Environmental	–	RT Anaesthetic Machine	*Klebsiella pneumoniae*
K45		EH	17/03/2016	Environmental	–	ICU Stocks	*Klebsiella pneumoniae*
K46		SAH1	29/07/2019	Clinical	Dog	Blood	*Klebsiella pneumoniae*
K47		SAH1	29/07/2019	Clinical	Dog	Abdominal fluid	*Klebsiella pneumoniae*
K48		SAH1	16/07/2019	Clinical	Dog	Skin lesion	*Klebsiella pneumoniae*
K49		SAH1	15/10/2018	Environmental	–	Treatment room tap	*Klebsiella pneumoniae*
K50		SAH1	15/10/2018	Environmental	–	Surgical ward tap	*Klebsiella pneumoniae*
K51		EH	08/08/2018	Clinical	Horse	Surgical site	*Klebsiella pneumoniae*
K52		SAH1	30/07/2018	Environmental	–	ICU Kennel	*Klebsiella pneumoniae*
K53		SAH1	23/07/2018	Clinical	Dog	Surgical site	*Klebsiella pneumoniae*
K54		SAH1	07/11/2017	Clinical	Dog	(Ortho) Surgical site?	*Klebsiella pneumoniae*
K55		SAH1	23/09/2017	Clinical	Dog	Urine	*Klebsiella pneumoniae*
K56		SAH1	14/09/2017	Clinical	Dog	Faeces	*Klebsiella pneumoniae*
K57		SAH1	12/09/2017	Clinical	Dog	Urine	*Klebsiella pneumoniae*
K58		SAP	23/07/2017	Clinical	Dog	Wound	*Klebsiella pneumoniae*
K59		SAH1	09/03/2017	Environmental	–	ICU Bandage Trolley	*Klebsiella pneumoniae*
K60		SAH1	07/03/2017	Clinical	Dog	Urine	*Klebsiella pneumoniae*
K61		EH	04/01/2017	Clinical	Horse	Surgical site	*Klebsiella pneumoniae*
K62		SAH1	07/12/2016	Clinical	Dog	PEG Tube Site	*Klebsiella pneumoniae*
K63		SAH1	05/10/2016	Clinical	Dog	Skin lesion	*Klebsiella pneumoniae*
K64		SAH1	26/01/2016	Environmental	–	Ward shower head	*Klebsiella pneumoniae*
K65		SAP	10/09/2015	Clinical	Dog	Urine	*Klebsiella pneumoniae*
K66		SAP	06/08/2015	Clinical	Cat	Fresh tissue	*Klebsiella pneumoniae*
K67		SAP	01/08/2015	Clinical	Cat	Wound	*Klebsiella pneumoniae*
K68		SAP	25/07/2015	Clinical	Dog	Wound	*Klebsiella pneumoniae*
K69		SAP	16/07/2015	Clinical	Dog	Fresh tissue	*Klebsiella pneumoniae*
K70		SAP	19/06/2015	Clinical	Dog	Skin lesion	*Klebsiella pneumoniae*
K71		SAP	27/05/2015	Clinical	Dog	Urine	*Klebsiella pneumoniae*
K72		EH	24/04/2015	Clinical	Horse	Surgical Site	*Klebsiella pneumoniae*
K73		EH	17/04/2015	Environmental	–	Stable A3	*Klebsiella pneumoniae*
K74		EH	22/12/2014	Clinical	Horse	Surgical site	*Klebsiella pneumoniae*
K75		EH	27/06/2014	Clinical	Horse	Surgical Site	*Klebsiella pneumoniae*
K76		SAH1	17/01/2014	Clinical	Dog	Surgical site	*Klebsiella pneumoniae*
K77		SAH1	14/10/2013	Clinical	Dog	Urine	*Klebsiella pneumoniae*
K78		SAH1	10/10/2013	Clinical	Dog	Urine	*Klebsiella pneumoniae*
K79		SAH1	09/10/2013	Clinical	Dog	Wound	*Klebsiella pneumoniae*
K80		SAH1	31/08/2013	Clinical	Dog	Thoracic fluid	*Klebsiella pneumoniae*
K81		SAH1	15/08/2013	Clinical	Dog	Skin lesion	*Klebsiella pneumoniae*
K82		EH	12/08/2013	Environmental	–	Stable A8	*Klebsiella pneumoniae*
K83		EH	29/06/2013	Environmental	–	Y-piece no. 1	*Klebsiella pneumoniae*
K84		EH	28/06/2013	Environmental	–	ICU Stocks	*Klebsiella pneumoniae*
K85		EH	21/06/2013	Environmental	–	Stable D6 Floor	*Klebsiella pneumoniae*
K86		EH	06/06/2013	Clinical	Horse	Rectal Swab	*Klebsiella pneumoniae*
K87		SAH1	21/06/2012	Clinical	Dog	ET tube swab	*Klebsiella pneumoniae*
K88		SAH2	04/07/2019	Environmental	–	ICU Air con vents pre-clean	*Klebsiella pneumoniae*
K89		SAH2	04/07/2019	Environmental	–	ICU Kennel keys/drawers pre-clean	*Klebsiella pneumoniae*
K90		SAH2	04/07/2019	Environmental	–	ICU Air con vents post-clean	*Klebsiella pneumoniae*
K91		SAH2	04/07/2019	Environmental	–	ICU Kennel keys/drawers post-clean	*Klebsiella pneumoniae*
K92		SAH2	04/07/2019	Environmental	–	ICU Tap & sink post-clean	*Klebsiella pneumoniae*

ICU PS, Intensive Care Unit Pilot Study; SAH1, Small Animal Hospital 1 (University of Liverpool); EH, Equine Hospital (University of Liverpool); SAP, Small Animal Practice (external); SAH2, Small Animal Hospital 2 (external).

**Table 2:** Sample type and origin of clinical, faecal and environmental MDR *Pseudomonas aeruginosa* (*n* = 107) from three companion animal veterinary facilities in the UK (2016–2019).

Isolate ID	ICU PS	Hospital	Date collected	Sample type	Animal species	Site	Isolate
P1	Yes	EH	20/03/2018	Faecal	Horse	ICU	*Pseudomonas aeruginosa*
P2		SAH1	20/03/2018	Environmental	–	ICU Keyboard	*Pseudomonas aeruginosa*
P3		SAH1	20/03/2018	Environmental	–	ICU Floor	*Pseudomonas aeruginosa*
P4		SAH1	20/03/2018	Environmental	–	ICU Phone	*Pseudomonas aeruginosa*
P5		SAH1	20/03/2018	Environmental	–	ICU Phone	*Pseudomonas aeruginosa*
P6		EH	23/03/2018	Faecal	Horse	ICU	*Pseudomonas aeruginosa*
P7		SAH1	04/04/2018	Environmental	–	ICU Floor	*Pseudomonas aeruginosa*
P8		EH	20/04/2018	Faecal	Horse	ICU	*Pseudomonas aeruginosa*
P9		EH	30/04/2018	Faecal	Horse	ICU	*Pseudomonas aeruginosa*
P10		EH	03/05/2018	Faecal	Horse	ICU	*Pseudomonas aeruginosa*
P11		EH	03/05/2018	Environmental	–	ICU Window	*Pseudomonas aeruginosa*
P12		EH	09/05/2018	Faecal	Horse	ICU	*Pseudomonas aeruginosa*
P13		EH	09/05/2018	Environmental	–	ICU Window	*Pseudomonas aeruginosa*
P14		EH	09/05/2018	Environmental	–	ICU Water Bucket	*Pseudomonas aeruginosa*
P15		EH	14/05/2018	Environmental	–	ICU Hay Rack	*Pseudomonas aeruginosa*
P16		EH	14/05/2018	Faecal	Horse	ICU	*Pseudomonas aeruginosa*
P17		EH	14/05/2018	Environmental	–	ICU Water Bucket	*Pseudomonas aeruginosa*
P18		EH	16/05/2018	Environmental	–	ICU Hay Rack	*Pseudomonas aeruginosa*
P19		EH	16/05/2018	Environmental	–	ICU Water Bucket	*Pseudomonas aeruginosa*
P20		EH	16/05/2018	Faecal	Horse	ICU	*Pseudomonas aeruginosa*
P21		EH	16/05/2018	Environmental	–	ICU Window	*Pseudomonas aeruginosa*
P22		EH	16/05/2018	Environmental	–	ICU Front Door Handle	*Pseudomonas aeruginosa*
P23		EH	16/05/2018	Environmental	–	ICU Hay Rack	*Pseudomonas aeruginosa*
P24		EH	17/05/2018	Faecal	Horse	ICU	*Pseudomonas aeruginosa*
P25		EH	17/05/2018	Environmental	–	ICU Window	*Pseudomonas aeruginosa*
P26		EH	17/05/2018	Environmental	–	ICU Hay Rack	*Pseudomonas aeruginosa*
P27		EH	17/05/2018	Environmental	–	ICU Tie Ring	*Pseudomonas aeruginosa*
P28		EH	17/05/2018	Faecal	Horse	ICU	*Pseudomonas aeruginosa*
P29		EH	17/05/2018	Environmental	–	ICU Window	*Pseudomonas aeruginosa*
P30		EH	17/05/2018	Environmental	–	ICU Water Bucket	*Pseudomonas aeruginosa*
P31		EH	21/05/2018	Faecal	Horse	ICU	*Pseudomonas aeruginosa*
P32		EH	21/05/2018	Environmental	–	ICU Water Bucket	*Pseudomonas aeruginosa*
P33		EH	22/05/2018	Faecal	Horse	ICU	*Pseudomonas aeruginosa*
P34		EH	22/05/2018	Environmental	–	ICU Water Bucket	*Pseudomonas aeruginosa*
P35		SAH1	22/05/2018	Environmental	–	ICU Door plate	*Pseudomonas aeruginosa*
P36		SAH1	22/05/2018	Environmental	–	ICU Floor	*Pseudomonas aeruginosa*
P37	No	SAH1	10/10/2016	Environmental	–	Chemotherapy ICU Kennel Handle	*Pseudomonas aeruginosa*
P38		SAH1	26/10/2016	Environmental	–	Wards Drip Pump Holder	*Pseudomonas aeruginosa*
P39		SAH1	01/08/2016	Environmental	–	Wards Keyboard	*Pseudomonas aeruginosa*
P40		SAH1	01/08/2016	Environmental	–	ICU Door Handle	*Pseudomonas aeruginosa*
P41		EH	13/11/2017	Clinical	Horse	Wound	*Pseudomonas aeruginosa*
P42		SAH1	2017	Environmental	–	Congregation water dispenser tray	*Pseudomonas aeruginosa*
P43		EH	23/06/2016	Environmental	–	Stable A1	*Pseudomonas aeruginosa*
P44		SAH1	15/06/2018	Environmental	–	Sink tap	*Pseudomonas aeruginosa*
P45		SAH1	01/05/2018	Clinical	Dog	Surgical Site	*Pseudomonas aeruginosa*
P46		SAH1	20/03/2018	Clinical	Dog	Wound	*Pseudomonas aeruginosa*
P47		EH	17/03/2016	Environmental	–	Laboratory Worktops	*Pseudomonas aeruginosa*
P48		SAH1	01/12/2017	Environmental	–	Dermatology Keyboard	*Pseudomonas aeruginosa*
P49		SAH1	30/03/2016	Environmental	–	Wards Shower Head	*Pseudomonas aeruginosa*
P50		EH	17/03/2016	Environmental	–	ICU Stocks	*Pseudomonas aeruginosa*
P51		SAH1	11/09/2019	Clinical	Cat	Wound	*Pseudomonas aeruginosa*
P52		SAH1	09/09/2019	Clinical	Dog	O’ tube site	*Pseudomonas aeruginosa*
P53		SAH1	06/09/2019	Environmental	–	Anaesthesia breathing system	*Pseudomonas aeruginosa*
P54		SAH1	20/08/2019	Clinical	Dog	Traumatic wound	*Pseudomonas aeruginosa*
P55		SAH1	13/08/2019	Environmental	–	Theatre black crocs	*Pseudomonas aeruginosa*
P56		SAH1	24/06/2019	Clinical	Dog	Surgical wound	*Pseudomonas aeruginosa*
P57		SAH1	24/06/2019	Clinical	Dog	Surgical wound	*Pseudomonas aeruginosa*
P58		SAH1	04/06/2019	Clinical	Dog	Urine	*Pseudomonas aeruginosa*
P59		SAH1	02/04/2019	Environmental	–	Treatment room table	*Pseudomonas aeruginosa*
P60		SAH1	02/04/2019	Environmental	–	Washroom hairdryer	*Pseudomonas aeruginosa*
P61		SAH1	27/03/2019	Environmental	–	Lab keyboard	*Pseudomonas aeruginosa*
P62		SAH1	25/01/2019	Clinical	Dog	External fixator discharge	*Pseudomonas aeruginosa*
P63		SAH1	22/01/2019	Environmental	–	Wards washing machine door	*Pseudomonas aeruginosa*
P64		SAH1	22/01/2019	Environmental	–	Treatment room tap before cleaning	*Pseudomonas aeruginosa*
P65		SAH1	22/01/2019	Environmental	–	Treatment room tap after cleaning	*Pseudomonas aeruginosa*
P66		SAH1	18/01/2019	Clinical	Dog	Interdigital swab post-surgical	*Pseudomonas aeruginosa*
P67		SAH1	16/01/2019	Environmental	–	Wards washing machine	*Pseudomonas aeruginosa*
P68		SAH1	16/01/2019	Environmental	–	Treatment room tap 1	*Pseudomonas aeruginosa*
P69		SAH1	03/01/2019	Clinical	Dog	Synovial fluid post-surgical	*Pseudomonas aeruginosa*
P70		SAH1	30/10/2018	Clinical	Dog	Ortho implants post-TPLO	*Pseudomonas aeruginosa*
P71		SAH1	30/10/2018	Clinical	Dog	Synovial fluid - Stifle	*Pseudomonas aeruginosa*
P72		SAH1	15/10/2018	Environmental	–	Washing machine door (wards)	*Pseudomonas aeruginosa*
P73		SAH1	14/09/2018	Environmental	–	Surgical ward tap	*Pseudomonas aeruginosa*
P74		SAH1	12/10/2018	Clinical	Dog	Synovial fluid post-TPLO	*Pseudomonas aeruginosa*
P75		SAH1	30/08/2018	Environmental	–	ICU Kennel	*Pseudomonas aeruginosa*
P76		SAH1	30/07/2018	Environmental	–	Treatment room	*Pseudomonas aeruginosa*
P77		SAH1	15/06/2018	Environmental	–	Sink tap	*Pseudomonas aeruginosa*
P78		SAH1	31/07/2017	Clinical	Dog	Surgical wound	*Pseudomonas aeruginosa*
P79		SAH1	13/07/2017	Clinical	Dog	External otitis	*Pseudomonas aeruginosa*
P80		SAH1	07/07/2017	Clinical	Dog	External otitis	*Pseudomonas aeruginosa*
P81		SAH1	28/06/2017	Clinical	Dog	Skin disease	*Pseudomonas aeruginosa*
P82		SAH1	21/06/2017	Clinical	Dog	Biopsy (fresh tissue)	*Pseudomonas aeruginosa*
P83		SAH1	09/06/2017	Clinical	Dog	External otitis	*Pseudomonas aeruginosa*
P84		SAH1	08/06/2017	Clinical	Cat	O-tube site	*Pseudomonas aeruginosa*
P85		SAH1	12/05/2017	Clinical	Dog	Skin swab	*Pseudomonas aeruginosa*
P86		SAH1	11/05/2017	Clinical	Cat	R frontal sinus fluid	*Pseudomonas aeruginosa*
P87		SAH1	10/04/2017	Clinical	Dog	Urine	*Pseudomonas aeruginosa*
P88		SAH1	17/03/2017	Clinical	Dog	Faeces	*Pseudomonas aeruginosa*
P89		SAH1	07/03/2017	Clinical	Dog	Urine	*Pseudomonas aeruginosa*
P90		SAH1	17/02/2017	Clinical	Dog	Tympanic bulla (fresh tissue)	*Pseudomonas aeruginosa*
P91		SAH1	02/02/2017	Clinical	Dog	External otitis	*Pseudomonas aeruginosa*
P92		SAH1	02/02/2017	Clinical	Dog	ET tube swab	*Pseudomonas aeruginosa*
P93		SAH1	13/02/2017	Clinical	Dog	Stifle wound swab	*Pseudomonas aeruginosa*
P94		SAH1	30/01/2017	Clinical	Dog	External otitis	*Pseudomonas aeruginosa*
P95		SAH1	23/01/2017	Clinical	Dog	External otitis	*Pseudomonas aeruginosa*
P96		SAH1	23/01/2017	Clinical	Dog	L tympanic bulla post-flush	*Pseudomonas aeruginosa*
P97		SAH1	11/01/2017	Clinical	Dog	Urine	*Pseudomonas aeruginosa*
P98		SAH1	11/04/2017	Clinical	Dog	Urine	*Pseudomonas aeruginosa*
P99		SAH1	07/02/2017	Environmental	–	Tea room tap	*Pseudomonas aeruginosa*
P100		SAH1	22/01/2017	Environmental	–	Washroom cupboard	*Pseudomonas aeruginosa*
P101		SAH1	2017	Clinical	Dog	Wound swab	*Pseudomonas aeruginosa*
P102		SAH2	11/07/2019	Environmental	–	ICU pre-clean desk area	*Pseudomonas aeruginosa*
P103		SAH2	11/07/2019	Environmental	–	ICU pre-clean cleaning cupboard	*Pseudomonas aeruginosa*
P104		SAH2	11/07/2019	Environmental	–	ICU pre-clean tap & sink	*Pseudomonas aeruginosa*
P105		SAH2	11/07/2019	Environmental	–	ICU pre-clean floor & drain	*Pseudomonas aeruginosa*
P106		SAH2	11/07/2019	Environmental	–	ICU post-clean cleaning cupboard	*Pseudomonas aeruginosa*
P107		SAH2	11/07/2019	Environmental	–	ICU post-clean floor & drain	*Pseudomonas aeruginosa*

ICU PS, Intensive Care Unit Pilot Study; SAH1, Small Animal Hospital 1 (University of Liverpool); EH, Equine Hospital (University of Liverpool); SAH2, Small Animal Hospital 2 (external).

In addition, MDR *K. pneumoniae* (*n* = 48) and *P. aeruginosa* (*n* = 65) isolated through routine diagnostics (RD) of clinical (CL) and environmental (ENV) specimens from the same UoL hospitals, were included in the analysis. These isolates covered a broader range of patients and hospital areas than the ICUs, and were collected from 2012 to 2019 for *K. pneumoniae* (Table 1) and from 2016 to 2019 for *P. aeruginosa* (Table 2). Furthermore, several isolates (*n* = 13 *K. pneumoniae* and *n* = 6 *P. aeruginosa*) received in the microbiology laboratory from two external small animal facilities (one hospital [SAH2] and one practice [SAP]) for environmental surveillance (*n* = 11) or for routine clinical diagnostics (*n* = 8) were also included, resulting in *n* = 199 bacterial isolates analysed overall with FTIR spectroscopy, whose summary is presented in Table 3.

**Table 3:** Summary of *Klebsiella pneumoniae* and *Pseudomonas aeruginosa* isolates (*n =* 199) from veterinary settings (small animal and equine) analysed by Fourier-transform Infrared (FTIR) spectroscopy.

	*Klebsiella pneumoniae*	*Pseudomonas aeruginosa*
Small animal		
ICU-PS	31	7
Faecal	5	0
Environmental	26	7
RD	46	67
Clinical	29	36
Environmental	17	31
Equine		
ICU-PS	0	29
Faecal	–	12
Environmental	–	17
RD	15	4
Clinical	6	1
Environmental	9	3
Total	92	107

ICU-PS, Intensive Care Unit Pilot Study, including faecal colonisation and environmental isolates; RD, Routine diagnostics, including clinical and environmental surveillance isolates from the University of Liverpool (SAH1 and EH) and two external facilities (SAH2 and SAP).

### 2.2 Sample processing

Clinical and environmental specimens processing was performed as previously described (Zendri et al., 2023). In brief, clinical isolates were obtained through routine microbiology diagnostics, processed in accordance with local laboratory protocols for culture and antimicrobial susceptibility testing (AST) using the CLSI methodology and interpretative criteria (Clinical and Laboratory Standards Institute (CLSI), 2018). All isolates were identified using MALDI-TOF MS (MALDI Biotyper 4.1.100 Software, Bruker Daltonics, Bremen, Germany) with a score > 2.0. Hospital environmental bacterial isolates were obtained from routine environmental surveillance samples, collected aseptically by veterinary nurses using sterile gauzes placed individually in Buffered Peptone Water (BPW) and enriched overnight at 37°C aerobically. To screen for MDR isolates, a small BPW inoculum in which faeces and ENV samples were placed, was plated onto eosin methylene blue agar (EMBA; Thermo Scientific) containing 1 mg/mL of cefotaxime (Sigma-Aldrich Ltd., United Kingdom) and incubated for 24 h at 37°C aerobically. EMBA negative morphotypes identified as *Klebsiella pneumoniae* and *Pseudomonas aeruginosa* using MALDI-TOF MS were included in the downstream analysis. All environmental surveillance and clinical MDR isolates were continuously stored at-80°C in the local strain collection; inclusion of these isolates in the study was based on phenotypic resistance to extended-spectrum cephalosporins and MDR status upon broth microdilution and/or disc diffusion antimicrobial susceptibility testing (AST) performed at the time of diagnostics.

### 2.3 Fourier-transform infrared spectroscopy

A total of *n =* 199 MDR bacterial isolates, of which *n* = 92 *K. pneumoniae* and *n* = 107 *P. aeruginosa*, from all hospitals/clinics were typed using FTIR spectroscopy (IR Biotyper, Bruker Daltonics, Bremen, Germany) to determine their clonal relatedness. Bacterial isolates were grown on 5% sheep Blood Agar under strictly controlled temperature, time and humidity conditions (22 h incubation at 37°C under aerobic atmosphere) and harvested in Eppendorf tubes to create bacterial suspensions in equal parts of 70% ethanol and molecular grade water before spotting the target plate. Tests were run with three technical replicates in each of three independent experiments with two bacterial standards for internal quality control included in each run. Spectra were acquired in transmission mode using the OPUS v7.5 software (Bruker Optics GmbH). Spectra were then pre-processed (calculated 2nd derivative, cut to 1,300–800 cm-1, vector-normalised) and further analysed with the IR Biotyper Client Software v1.5 (Bruker Daltonics GmbH). Dendrograms expressing Hierarchical Cluster Analysis (HCA) were generated by the IR Biotyper Client Software v1.5 using the Euclidean distance and average linkage clustering methods using Cut-Off Values (COVs) to define clusters of 0.246 and of 0.287 for *P. aeruginosa* and *K. pneumoniae*, respectively. An optimisation procedure was conducted beforehand to determine optimal COVs for these organisms that were specific to our local laboratory conditions. The isolates used for species-specific optimisation of the COVs included epidemiologically related and non-related (e.g., reference strains) small animal *K. pneumoniae* and *P. aeruginosa* from external sources. The COVs were established according to manufacturer’s recommendations on three repetitive experiments each using five technical and/or biological replicates of *K. pneumoniae* (*n =* 8) and *P. aeruginosa* (*n =* 9) isolates, previously typed by MLST or WGS. The automatically generated COVs were adjusted on the dendograms based on prior knowledge of the genetic relatedness of the *K. pneumoniae* and *P. aeruginosa* subsets of isolates used for the optimisation process.

### 2.4 Whole-genome sequencing and bioinformatics

To evaluate the performance of FTIR spectroscopy against a gold standard typing method, a subset of isolates demonstrating clonal relatedness was further characterised by WGS. Fifty-four *P. aeruginosa* and 27 *K. pneumoniae* isolates were selected, covering most FTIR clusters in a proportional measure to the cluster size. Fragment libraries (NEBNext Ultra II FS Kit; ∼300 base pair inserts) were created from purified genomic DNA (Qiagen QIAmp DNA mini kit) and sequenced using a 2 × 150 base pair paired-end protocol (Illumina NovaSeq SP; CGR, University of Liverpool). *De novo* assembly was performed using SPAdes 3.16.0 (Bankevich et al., 2012) and genome annotation via Prokka 1.14.5 (Seemann, 2014). MLST profiles and allele sequences were obtained.^1^ All allele sequences were aligned to assemblies using Bowtie2 version 2.3.5.1 (Langmead and Salzberg, 2012) in sensitive mode. The allele that aligned best for each locus was selected and the sequence type was determined by comparing perfectly detected alleles against the database profiles. Sequence types were used to infer eBURST groups, using goeBURST,^2^ where group members shared at least 2 ST locus alleles. *In silico* identification of virulence and AMR genes was performed using the Virulence Factor Database (VFDB; Chen et al., 2005) and the Comprehensive Antibiotic Resistance Database (CARD; McArthur et al., 2013), respectively. Incompatibility (Inc) group plasmids were identified in *K. pneumoniae* via PlasmidFinder (Carattoli et al., 2014). Using assemblies as input, pangenome reconstruction and concatenated core gene multiple alignment was carried using Panaroo (Tonkin-Hill et al., 2020). Subsequently, the multiple alignment data was used as input to IQ-TREE using the GTR model. The resulting tree was imported to the Interactive Tree of Life (iTOL; Letunic and Bork, 2021). Genomic sequences are deposited on the European Nucleotide Archive (ENA) under the project accession: PRJEB70897.

### 2.5 Concordance between FTIR and MLST clusters

The clusters defined by MLST extrapolated from genomic sequences were compared with those obtained by FTIR biotyping using the service^3^ by calculating the Adjusted Rand index (AR) and Adjusted Wallace coefficient (AW) with 95% confidence (Carriço et al., 2006). AR compares partitions without consideration of the reference method and evaluates the congruence between two typing methods. In comparison, AW provides inter-cluster distances and evaluates the directional agreement between typing methods (Carriço et al., 2006; Pinto et al., 2008; Severiano et al., 2011a,b). In essence, AR and AW equal to 1 indicate a perfect correlation between the two typing methods. The optimal cut-offs for each species were defined by maximising AR between MLST and FTIR results.

## 3 Results

### 3.1 Fourier-transform infrared spectroscopy

After FTIR spectra acquisition, dendrograms were built for each species. FTIR analysis identified several clusters for both organisms tested, suggesting clonal relatedness of the isolates within individual clusters. Overall, *n =* 25 FTIR clusters were identified spanning across both pathogens; the number of isolates within each cluster varied, ranging from two to 43, with an average cluster size of 6.8 isolates. Fourteen of 25 clusters (56%) contained a mixture of environmental and clinical and/or faecal carriage isolates in various combinations, typically several environmental isolates linked to fewer clinical/carriage ones. Wound and skin isolates were the most common clinical specimen type represented within mixed clusters. Environmental isolates only, collected from different hospitals’ sites, also accounted for some of the clusters observed. Several isolates collected from the ICUs contributed to cluster formation, either with or without association to isolates collected from the broader hospital patients and departments. Details are provided in the following paragraphs for each organism.

#### 3.1.1 FTIR of *Klebsiella pneumoniae*

**Figure 1:**
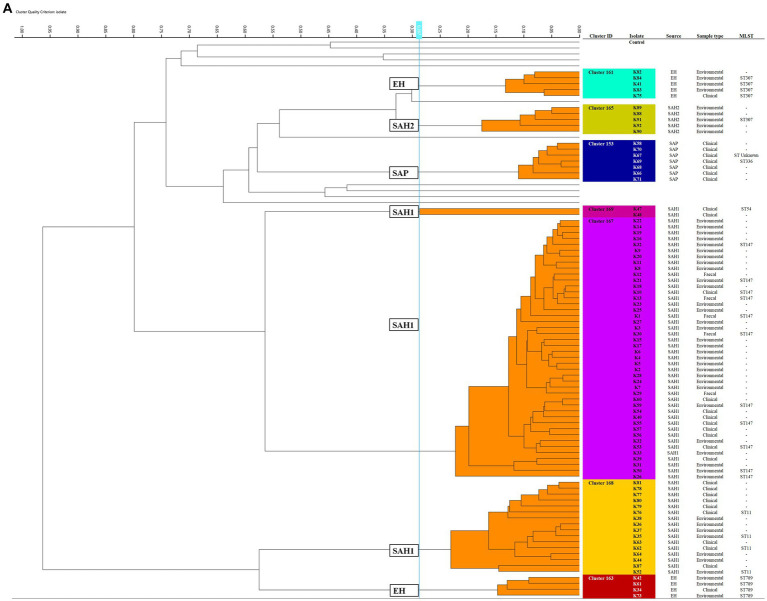

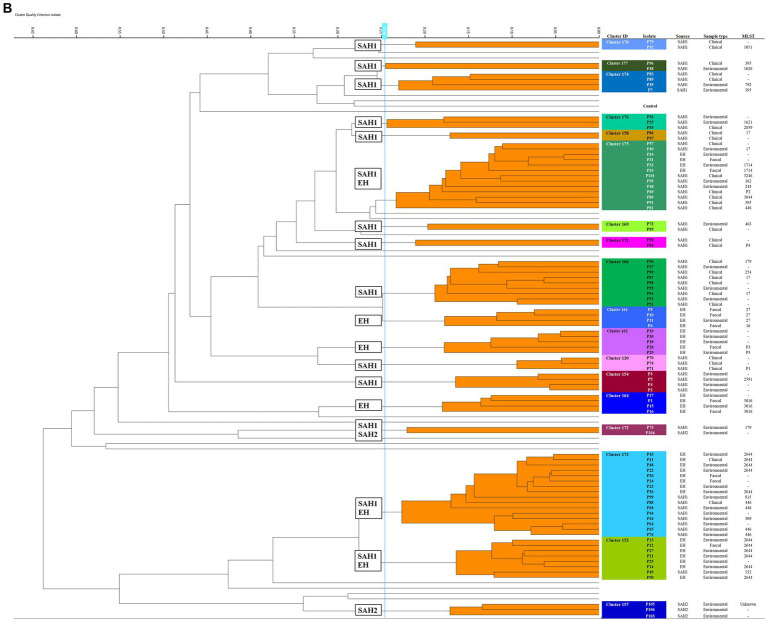
Fourier-transform Infrared (FTIR) biotyping of *Klebsiella pneumoniae* subsp. *pneumoniae* **(A)** and *Pseudomonas aeruginosa* **(B)** veterinary isolates. Dendograms were obtained by Euclidean average spectra analysis of *K. pneumoniae* (*n* = 92) and *P. aeruginosa* (*n* = 107) from clinical, faecal and environmental surveillance specimens collected in small animal and equine veterinary facilities. Analysis was based on three technical replicates of each isolate and included one control strain per each organism; the blue line represents the adjusted cut-off values (COVs) of 0.287 and 0.246 for *K. pneumoniae* and *P. aeruginosa*, respectively. Clusters comprising two or more isolates are shaded in orange. Isolate data is shown for clusters only. SAH1: Small Animal Hospital 1 (University of Liverpool); EH, Equine Hospital (University of Liverpool); SAP, Small Animal Practice (external); SAH2, Small Animal Hospital 2 (external).

Seven *K. pneumoniae* clusters were defined overall totalling 83/92 isolates (Figure 1A); of these, the largest (Cluster 167) contained 43 small animal isolates including environmental, faecal carriage and clinical isolates collected over a 19-month period (March 2017–October 2018); this cluster included isolates predominantly obtained from the SAH1 ICU during the PS (31/43) with fewer non-ICU isolates also found in this cluster. All isolates sequenced from this large cluster (*n* = 11) belonged to ST147. The ICU *K. pneumoniae* isolates from Cluster 167, were identified on numerous ICU surfaces and canine faecal carriage samples within a relatively short time-frame (31 isolates over 16 days). Contaminated environmental sites included ICU telephone receiver, computer keyboards, door handles, equipment trolley, floor and patients’ kennels. One clinical *K. pneumoniae* was cultured from a case of hospital-acquired septic peritonitis in a dog enrolled in the ICU study that was previously found to be a faecal carrier of the same strain. Sixteen isolates accounted for the second largest *K. pneumoniae* FTIR cluster (Cluster 168), comprising clinical and environmental isolates collected from the wider SAH1 environments over a 6-year period (from 2012 to 2018). Clinical isolates included in this group consisted of skin and wound isolates (*n* = 2 sequenced, ST11) but also sterile fluid ones (thoracic fluid and cystocentesis urine) alongside environmental isolates from clinical (e.g., anaesthetic machine and ICU surfaces; *n* = 2 sequenced, ST11) and non-clinical (e.g., reception photocopier, lockers) hospital areas.

Of note, all *K. pneumoniae* from external small animal settings accurately clustered with isolates from their respective hospital source (SAP for Cluster 153 and SAH2 for Cluster 165).

Two small clusters (of 5 and 4 isolates; Clusters 161 and 163) of environmental *K. pneumoniae* (e.g., from stables, ICU) in association with post-surgical infection isolates were identified in the EH; these belonged to ST307 and ST789, respectively.

Importantly, clonal dissemination of *K. pneumoniae* was identified in all veterinary settings; two clones were circulating in each one of the UoL hospitals (ST147 and ST11 in the SAH1 and ST307 and ST789 in the EH). In addition, single clones were identified in the two external facilities included in the study, i.e., Cluster 165 composed of environmental isolates from the SAH2 ICU (ST307) and Cluster 153 of clinical isolates originating from the SAP (ST336).

#### 3.1.2 FTIR of *Pseudomonas aeruginosa*

Of the 18 *P. aeruginosa* FTIR clusters, the two largest contained 16 (Cluster 173) and 13 (Cluster 175) mixed EH and SAH1 isolates collected over two distinct 3-year periods (Figure 1B). Cluster 173 comprised *P. aeruginosa* isolated from a horse faecal samples and multiple ICU environmental sites surrounding the patient (*n* = 2 sequenced, ST2644), alongside few other clinical and environmental isolates from the broader EH one to 2 years prior (*n* = 3 sequenced, ST2644). Small animal *P. aeruginosa* in Cluster 173 were mostly water-borne environmental isolates (mostly typed to ST446, but also ST309 and ST815) cultured from diverse sink taps within the SAH1 (e.g., treatment room taps, wards shower head and tea room tap) over a 3-year interval. Cluster 175 comprised *P. aeruginosa* isolates linked to a single ICU PS horse (*n* = 2 sequenced, ST1714) and the remainder from the SAH1. In this cluster, small animal isolates were mostly clinical *P. aeruginosa* from surgical wounds and otitis cases cultured between 2017 and 2019 (*n* = 8 sequenced, multiple STs). Small ICU clusters (4–6 isolates) of PS isolates (faecal carriage and environmental) linking 2–3 patients over short periods (from 2 days to 7 weeks) were established in the EH (Clusters 161, 162, 164 and 153) but not in the SAH1 (Cluster 154 was linked to a single patient). Some other clusters (2–8 isolates; Clusters 170, 174, 176, 177, 158, 169, 171, 166 and 130) comprised various combinations of environmental and/or clinical isolates within the SAH1; *P. aeruginosa* isolated from a range of clinical specimens featured in these clusters, including both normally contaminated (wound, skin and ear) and sterile (cystocentesis urine and synovial fluid) body sites.

### 3.2 Whole-genome sequencing and comparison with FTIR typing

In total, 81 (*n* = 81) isolates underwent WGS (at least one isolate per cluster and up to nine) and MLST, eBURST and other genotypic data were extracted from whole genomes. MLST, eBURST groups and AMR genes are summarised in Table 4 (full characterisation including virulence genes and plasmid types is found in Supplementary Table S1). Concordance between the FTIR and WGS findings was overall high for *K. pneumoniae* and poor for *P. aeruginosa* using the established COVs. For *K. pneumoniae*, both typing methods, FTIR and WGS, achieved the same level of discriminatory power, dividing the isolates into seven clusters each. The Simpson’s diversity index (SDI; Simpson, 1949; Hunter and Gaston, 1988) for both was 0.789 and 0.781, respectively. FTIR and WGS typing showed very high agreement, resulting in an adjusted Rand index (ARI; Carriço et al., 2006) of 0.958. The adjusted Wallace index (AWI; Severiano et al., 2011b) for FTIR predicting WGS types was 0.983, while in the reverse direction it was 0.934. For *P. aeruginosa*, the congruence of typing results was low. First, WGS was able to distinguish 28 different types (SDI 0.941) while FTIR could only distinguish 18 clusters (SDI 0.243). This was also reflected in quite asymmetrical AWIs, where FTIR predicted WGS types with 0.243, but WGS predicted FTIR clusters with 0.437. Overall congruence was 0.313 using the ARI.

**Table 4:** Comparison of Fourier-Transform InfraRed (FTIR) Spectroscopy and Multi-Locus Sequence Typing (MLST) results extracted from Whole-Genome Sequences (WGS).

WGS isolates (n)	WGS Isolate type (n)	Hospital source	WGS Isolate details	FTIR Cluster	Cluster isolation interval	ST Type	eBURST	AMR genes
*Klebsiella pneumoniae*								
4	CL (1), ENV (3)	EH	Surgical wound, stable, ICU stocks, Y piece	161	12 months	307	C4	*oqx*A/B, aac(6′)-Ib-cr, CTX-M-15, fosA6, SHV-28, TEM-206, *qnr*B
1	ENV	SAH2	Air con vent	165	1 day	307	C4	*oqx*A/B, CTX-M-15, fosA6, SHV-28, TEM-1, *qnr*B
2	CL	SAP	Biopsy, wound	153	26 months	336, UT^1^	C0, Unknown	*oqx*A/B,aac(6′)-Ib-cr, CTX-M-15, DHA-1, FosA6, SHV-11, TEM-1, *qnr*B
1	CL	SAH1	Blood	169	Single time point^2^	54	C1	*oqx*A/B, FosA5, SHV-11
11	ENV (5), *F* (3), CL (3)	SAH1	ICU: door handles, telephones, trolleys, faeces and abdominal fluid. Non-ICU: surgical ward tap, surgical wound, urine.	167	19 months	147	C5	*oqx*A/B, DHA-1, FosA6, SHV-11, *qnr*B
4	CL (2), ENV (2)	SAH1	Surgical wound, PEG tube site, ICU door handle and kennel	168	6 years	11	C2	*oqx*A/B,aac(6′)-Ib-cr, DHA-1, FosA2, FosA6, SHV-11, *qnr*B/S^3^
4	CL (1), ENV (3)	EH	Surgical wound, stables, student keyboard	163	21 months	789	C3	*oqx*A/B, FosA6, SHV-25, TEM-1, *qnr*S^4^
*Pseudomonas aeruginosa*								
1	CL	SAH1	Endotracheal tube	170	5 months	1,051	C29	MexAB-OprM, MexXY, MexCD-OprJ, MexEF-OprN, OXA-50, FosA, gyrA
1	CL	SAH1	Tympanic bulla fluid	177	3 months	395	C11	MexAB-OprM, MexXY, MexCD-OprJ, MexEF-OprN, OXA-50, FosA
1	ENV	SAH1	Wards Drip Pump Holder	177	3 months	1,026	C1	MexAB-OprM, MexXY, MexCD-OprJ, MexEF-OprN, OXA-50, FosA
1	ENV	SAH1	Wards keyboard	174	20 months	792	C8	MexAB-OprM, MexXY, MexCD-OprJ, MexEF-OprN, OXA-50, FosA
1	ENV	SAH1	ICU Floor	174	20 months	395	C11	MexAB-OprM, MexXY, MexCD-OprJ, MexEF-OprN, OXA-50, FosA
1	CL	SAH1	Skin	176	12 months	2,859	C7	MexAB-OprM, MexXY, MexCD-OprJ, MexEF-OprN, OXA-486, FosA
1	ENV	SAH1	ICU Door	176	12 months	1,621	C15	MexAB-OprM, MexXY, MexCD-OprJ, MexEF-OprN, OXA-50, FosA
1	CL	SAH1	Frontal sinus exudate	158	4 days	17	C20	MexAB-OprM, MexXY, MexCD-OprJ, MexEF-OprN, OXA-50, FosA
1	ENV	SAH1	ICU Door	175	3 years	17	C20	MexAB-OprM, MexXY, MexCD-OprJ, MexEF-OprN, OXA-50, FosA
2	F, ENV	EH	Faeces, ICU Water bucket	175	3 years	1714	C0	MexAB-OprM, MexXY, MexCD-OprJ, MexEF-OprN, OXA-50, FosA
1	CL	SAH1	Wound	175	3 years	3,246	C27	MexAB-OprM, MexXY, MexCD-OprJ, MexEF-OprN, OXA-50, FosA
1	ENV	SAH1	Treatment room table	175	3 years	362	C25	MexAB-OprM, MexXY, MexCD-OprJ, MexEF-OprN, OXA-50, FosA
1	ENV	SAH1	Dermatology keyboard	175	3 years	244	C4	MexAB-OprM, MexXY, MexCD-OprJ, MexEF-OprN, OXA-50, FosA
1	CL	SAH1	Synovial fluid	175	3 years	P2	C10	MexAB-OprM, MexXY, MexCD-OprJ, MexEF-OprN, OXA-50, FosA
1	CL	SAH1	External ear	175	3 years	3,044	C2	MexAB-OprM, MexXY, MexCD-OprJ, MexEF-OprN, OXA-50, FosA, gyrA
1	CL	SAH1	External ear	175	3 years	395	C11	MexAB-OprM, MexXY, MexCD-OprJ, MexEF-OprN, OXA-50, FosA
1	CL	SAH1	Skin	175	3 years	446	C12	MexAB-OprM, MexXY, MexCD-OprJ, MexEF-OprN, OXA-50, FosA
1	ENV	SAH1	Wards washing machine door	169	21 months	463	C9	MexAB-OprM, MexXY, MexCD-OprJ, MexEF-OprN, FosA
1	CL	SAH1	PEG tube wound	171	2 years	P4	C30	MexAB-OprM, MexXY, MexCD-OprJ, MexEF-OprN, OXA-50, FosA, qnrB
1	CL	SAH1	Wound	166	31 months	179	C23	MexAB-OprM, MexXY, MexCD-OprJ, MexEF-OprN, OXA-50, FosA
1	CL	SAH1	Tympanic bulla tissue	166	31 months	254	C22	MexAB-OprM, MexXY, MexCD-OprJ, MexEF-OprN, OXA-50, FosA
2	CL	SAH1	Urine, wound	166	31 months	17	C20	MexAB-OprM, MexXY, MexCD-OprJ, MexEF-OprN, OXA-50, FosA
3	*F* (2), ENV	EH	Faeces, ICU Window	161	41 days	27	C18	MexAB-OprM, MexXY, MexCD-OprJ, MexEF-OprN, OXA-50, FosA
1	F	EH	Faeces	161	41 days	16	C21	MexAB-OprM, MexXY, MexCD-OprJ, MexEF-OprN, OXA-50, FosA
2	F, ENV	EH	Faeces, ICU Window	162	2 days	P3	C0	MexAB-OprM, MexXY, MexCD-OprJ, MexEF-OprN, OXA-50, FosA
1	CL	SAH1	Synovial fluid	130	18 days	P1	C26	MexAB-OprM, MexXY, MexCD-OprJ, MexEF-OprN, OXA-50, FosA
1	ENV	SAH1	ICU phone	154	Single time point	2,591	C14	MexAB-OprM, MexXY, MexCD-OprJ, MexEF-OprN, OXA-50, FosA
3	F (2), ENV	EH	Faeces, ICU Hay rack	164	56 days	3,016	C6	MexAB-OprM, MexXY, MexCD-OprJ, MexEF-OprN, OXA-50, FosA
1	ENV	SAH1	Surgical ward tap	172	Unknown	179	C23	MexAB-OprM, MexXY, MexCD-OprJ, MexEF-OprN, OXA-50, FosA
4	ENV (4)	EH	ICU Door, ICU Hay rack, stable, laboratory worktop	173	2 years	2,644	C16	MexAB-OprM, MexXY, MexCD-OprJ, MexEF-OprN, FosA
1	CL	EH	Wound	173	2 years	2,644	C16	MexAB, MexXY, MexCD, MexEF, FosA
4	CL, ENV (3)	SAH1	Faeces, treatment room tap (3)	173	2 years	446	C12	MexAB-OprM, MexXY, MexCD-OprJ, MexEF-OprN, OXA-50, FosA
1	ENV	SAH1	Sink tap	173	2 years	309	C13	MexAB-OprM, MexXY, MexCD-OprJ, MexEF-OprN, OXA-50, FosA
1	ENV	SAH1	Tea room tap	173	2 years	815	C17	MexAB-OprM, MexXY, MexCD-OprJ, MexEF-OprN, OXA-50, FosA
6	F, ENV (5)	EH	Faeces, ICU window (2), water bucket, tie ring and stocks	153	26 months	2,644	C16	MexAB-OprM, MexXY, MexCD-OprJ, MexEF-OprN, FosA
1	ENV	SAH1	Wards shower head	153	26 months	532	C28	MexAB-OprM, MexXY, MexCD-OprJ, MexEF-OprN, FosA
1	ENV	SAH2	ICU floor and drain	157	Single time point	Unknown	Unknown	–

^1^Nearest ST: 336; ^2^Two samples collected from the same patient; ^3^aac(6′)-Ib-cr in 75%, FosA2 in 50% and qnrS in 25% of the isolates; ^4^qnrS in 75% of the isolates. SAH1, Small Animal Hospital 1 (University of Liverpool); EH, Equine Hospital (University of Liverpool); SAP: Small Animal Practice (external); SAH2, Small Animal Hospital 2 (external).

#### 3.2.1 *Klebsiella pneumoniae* WGS

**Figure 2:**
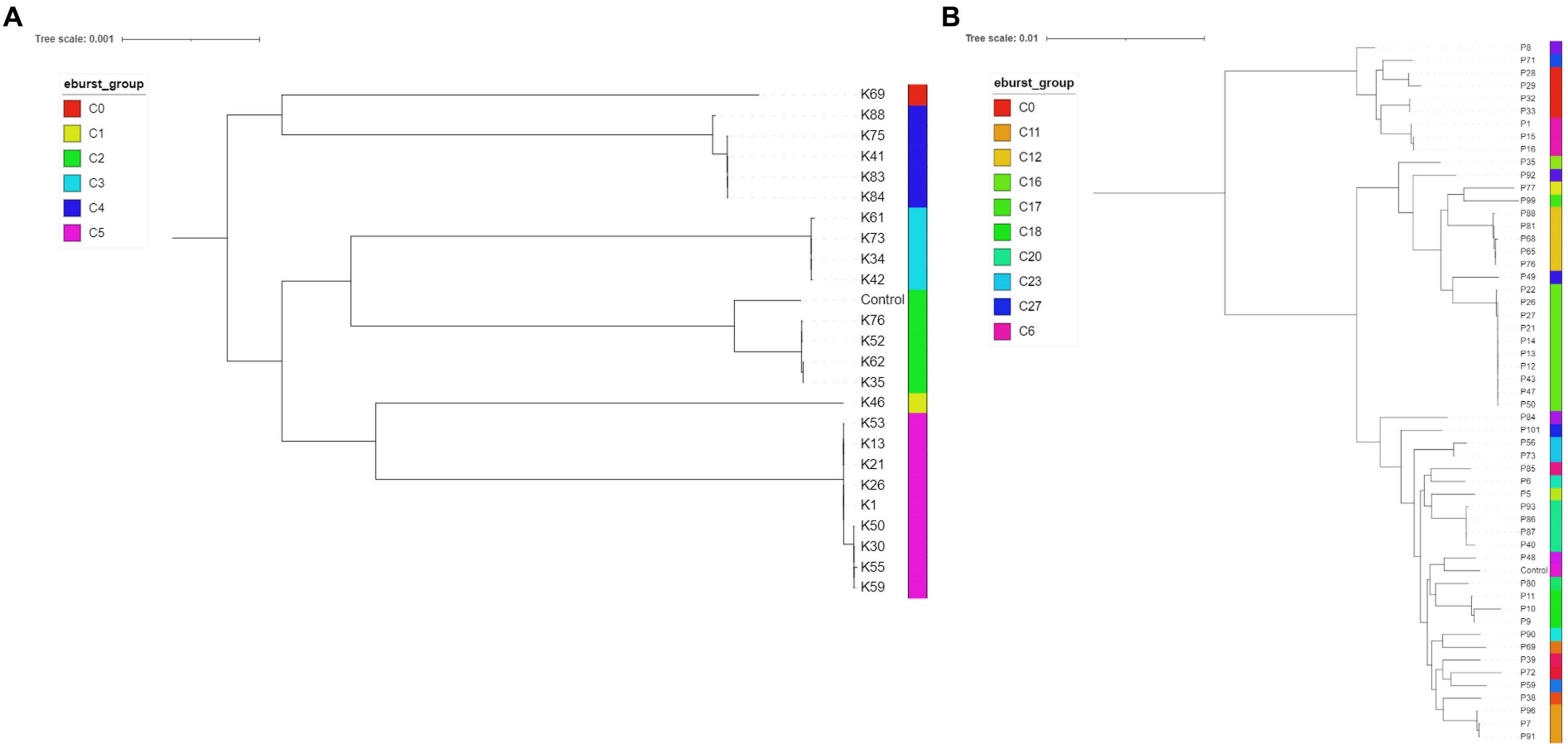
Midpoint rooted phylogenetic trees of *Klebsiella pneumoniae* (**A**, *n =* 24; isolates K10, K32 and K67 had incomplete core genome assembly, therefore they are not included in the below figure) and *Pseudomonas aeruginosa* (**B**, *n =* 53; P105 had incomplete genome assembly) isolates. The eBURST groups derived from MLST profiles extracted from the assembled genome sequences are shown. *Klebsiella pneumoniae* HS12286 and *Pseudomonas aeruginosa* PAO1 strains were used as internal controls.

Twenty-seven *K. pneumoniae* across the seven FTIR clusters had WGS data available. Results indicate high level agreement on the identification of related isolates between the spectroscopic and the genomic methods (Figures 1A, 2A); all sequenced *K. pneumoniae* isolates clustered by FTIR spectroscopy belonged to the same or closest ST (one isolate in Cluster 153 was of unknown ST type; however, the nearest ST match aligned with its cluster’s ST) with six STs identified overall. Notably, ST307 was detected in two adjacent Clusters, 161 and 165; the former gathering isolates from the EH while the latter from the SAH2, supporting the FTIR Biotyper ability to discriminate between isolates of the same sequence type. One important finding was the association of ST147 harbouring plasmid-encoded quinolone resistance genes *qnr*B and *oqx*A/B, SHV-11 and DHA-1 beta-lactamase types with Cluster 167, the largest FTIR cluster of *K. pneumoniae* intensely circulating in the SAH1 ICU which included the hospital-acquired infection case in a dog. ST11 was associated to *K. pneumoniae* in Cluster 168, linking isolates from the wider SAH1 patients and areas, and additionally carried the fluoroquinolone acetylating aminoglycoside-(6)-N-acetyltransferase (*aac[6′]-Ib-cr*) gene. CTX-M-15-producing *K. pneumoniae* ST307 was identified in the former of two EH clusters (Cluster 161) and *qnrS*-positive ST789 in the latter (Cluster 163). Beyond beta-lactamase resistance genes present in all isolates, multidrug efflux pump genes *oqxA/B* were detected in 100% (27/27) of sequenced *K. pneumoniae*, *qnr* and *aac(6′)-Ib-cr* genes mediating resistance to fluoroquinolones were identified in 81.8% (25/27) and 29.6% (8/27) of the sequenced *K. pneumoniae* isolates, respectively, while CTX-M-15 type enzyme in 22% (6/27) of isolates. IncR type plasmid was common among ST147 and ST11 while IncFIB and IncFII plasmids were both found among ST307 and ST336. Concordance between AMR genes (Clusters 168 and 163) and Inc. types (Clusters 161, 167, 168 and 163) among sequenced isolates was not absolute within some clusters (Table 4), as some but not all (generally ≥50%) isolates carried several certain determinants, such as *qnrS* and IncFIA in 3/4 of Cluster 163 isolates.

#### 3.2.2 *Pseudomonas aeruginosa* WGS

Fifty-four *P. aeruginosa* spanning over 18 FTIR clusters underwent WGS; in contrast to *K. pneumoniae*, the accuracy of FTIR clusters was generally poor when compared to WGS (Figures 1B, 2B). Heterogeneity of MLST types was observed in the vast majority of *P. aeruginosa* clusters where more than one isolate was sequenced, with up to nine STs detected in Cluster 175 (where 10 of 13 were sequenced). Multiple STs were also identified in Clusters 173 (*n =* 4 STs over 11 sequenced isolates) and 153 (*n =* 2 STs over 7 sequenced isolates), others combining SAH1 and EH isolates as Cluster 175. In all these mixed clusters, the equine isolates typically accounted for a single ST; notably, the majority of ST2644 isolates from Clusters 173 and 153 were isolated from EH ICU patients (faecal carriage) and surroundings during the pilot study over a short time. In contrast, the SAH1 isolates from any of the mixed clusters were of discordant ST (when more than one small animal isolate was found), apart from ST446 in Cluster 173 (here two thirds of sequenced small animal isolates belonged to this ST). Cluster 162 and 164 (*n =* 2 and 3 isolates sequenced, respectively), both comprising EH ICU pilot study isolates, were the only examples of genomically highly similar isolates appropriately clustered by the FTIR. SAH1 only clusters where more than one isolate was sequenced displayed substantial within-cluster ST heterogeneity, such as Clusters 166, 174 and 176. In addition to genomically divergent isolates inadequately clustered together by the FTIR, examples of genotypically highly similar isolates inappropriately not clustered were also seen, e.g., ST2644, ST395, ST17 and ST446 isolates from the same facility are seen across more than one cluster (usually 2 or 3). Finally, *P. aeruginosa* STs associated to clusters where single isolates were sequenced differed from one another and from STs in larger clusters, except for ST17. Overall, 28 STs were identified with ST2644, ST446, ST17, ST395, ST27 and ST3016 being most represented (Table 4). Significantly less variability was observed among *P. aeruginosa* with regards to AMR determinants. Sequenced isolates harboured a large arsenal of efflux pumps, particularly of the Mex (A/B, C/D, E/F, X/Y, M/J, and X/Y types amongst others) and Opr (OprJ, M and N) families and 73.7% contained OXA-50 (42/57) beta-lactamase; *gyrA* mutation and *qnrB* gene conferring fluoroquinolone resistance were detected in 5.3% (3/57) and 1.8% (1/57) of isolates, respectively.

## 4 Discussion

To the best of the authors’ knowledge, this is the first study to evaluate the FTIR potential for bacterial strain typing in a clinically-relevant timeframe within veterinary settings. We assessed the discriminatory power of FTIR spectroscopy by typing two ESKAPE MDR gram-negative bacterial species with potential implication in HCAIs, namely *K. pneumoniae* and *P. aeruginosa*, isolated from cases of clinical infection in hospitalised or vet-visiting companion animals (i.e., dogs, cats and horses), as well as faecal colonisation and environmental sites across veterinary clinics/hospitals. The IR Biotyper could determine clonal relatedness of MDR gram-negative isolates circulating in veterinary hospitals within 2–3 h from fresh culture harvest, making it an attractive tool for rapid strain typing of organisms associated with HCAIs. This technique has the potential to be integrated into the routine diagnostic workflow of a clinical microbiology laboratory for “real-time” monitoring of MDR pathogens’ transmission events to support prompt and targeted infection control measures during hospital outbreaks.

In our study, we found that the discriminatory power of FTIR spectroscopy was high for *K. pneumoniae* veterinary isolates using a COV of 0.287, which demonstrated overall concordance (ARI of 0.958) with WGS results for the fraction of isolates that underwent the gold standard technique. This is in agreement with previous studies evaluating the IR Biotyper performance on *K. pneumoniae*-associated human hospital outbreaks (Rakovitsky et al., 2020; Rodrigues et al., 2020; Silva et al., 2020; Hu et al., 2021). In one study assessing the IR Biotyper performance on environmental isolates of *K. pneumoniae* and other gram-negative bacilli from nine human hospitals, however, the instrument displayed limited sensitivity to cluster isolates compared with WGS (Aranega Bou et al., 2023); this contrasts with our findings where both environmental and clinical/carriage *K. pneumoniae* were adequately clustered according to WGS data. In addition, some authors evaluated the performance of the FTIR as a first-line typing tool for the identification of ESBL-producing *Klebsiella pneumoniae* outbreaks in the hospital setting in comparison with conventional epidemiology (CE) investigations and found both AR and AW were significantly higher for FTIR clustering than CE clustering (Wang-Wang et al., 2022). Importantly, the majority of *K. pneumoniae* isolates circulating in veterinary hospitals in our study were consistent with critically important human lineages and they segregated into well-defined hospital source clusters. Clonally related *K. pneumoniae* ST147 formed the largest SAH1 cluster comprising 72% of isolates collected from the ICU over 16 days. This lineage was identified simultaneously in carriage (faeces), environmental (patients kennels, ICU door handles, keyboards and phone) and one clinical infection case (septic peritonitis) in an ICU hospitalised dog. *K. pneumoniae* ST147 is an emerging high-risk human clone with worldwide distribution (Navon-Venezia et al., 2017) and the potential to become a major public health threat due to its ability to cause serious infections and association with AMR, including pan-resistance. This successful clone consists of multiple clades/clusters, often encoding for ESBLs and carbapenemases such as CTX-M-15, KPC-2 and NDM-1 amongst others (Peirano et al., 2020). To date, *K. pneumoniae* ST147 has been occasionally described in companion and exotic pets’ clinical specimens (Ovejero et al., 2017; Marques et al., 2019; Davies et al., 2022) without association to carbapenem resistance. Similar considerations may be formulated for *K. pneumoniae* ST11 identified in the SAH1 and ST307 and ST789 isolated from the EH; these epidemic human-associated clones (except for ST789) have already been described in companion animals more extensively than ST147 (Harada et al., 2016; Loncaric et al., 2020; Garcia-Fierro et al., 2022), with reports of OXA-48-producing ST11 strains causing animal infection in some European countries (Pulss et al., 2018). In contrast, *K. pneumoniae* ST15 broadly reported among dogs, cats and horses (Haenni et al., 2012; Stolle et al., 2013; Ewers et al., 2014; Maeyama et al., 2018), was not detected in the present study. Altogether, these findings point towards the possibility of clonal transmission of *K. pneumoniae* within all veterinary facilities investigated, as outlined by the FTIR association of clusters (and STs) with their hospital sources and, for isolates within some clusters, by the short sample collection window (e.g., the SAH1 ICU isolates in Cluster 167). Furthermore, the tight clustering of clinical isolates from the SAP (Cluster 153) on one hand, and of the environmental isolates from the SAH2 (Cluster 165), also points towards cross-transmission within these facilities.

Compared to *K. pneumoniae*, FTIR-based clustering of *P. aeruginosa* veterinary isolates was much less congruent with WGS results; its ability to correctly detect and cluster isolates of a given ST using a COV of 0.246 was poor, as reflected by an ARI of 0.313, with multiple examples of genomically related isolates spread across different clusters and genomically divergent isolates incorrectly clustered together by the IR Biotyper. Nevertheless, comparable diagnostic resolution to that of current reference methods has been previously reported for this organism by other authors (Martak et al., 2019; Hu et al., 2023). In these studies analysing clinical *P. aeruginosa* isolates from human hospital outbreaks, optimal FTIR spectroscopy COVs varied for this organism; one publication reports the optimal range 0.184 to 0.374 (AR, 0.936; Martak et al., 2019) while the other determined a more stringent COV range of 0.132 to 0.173 (AR, 0.711; Hu et al., 2023). Both studies employed Mueller-Hinton (MH) agar medium to passage *P. aeruginosa* isolates undergoing FTIR Biotyping, and Hu et al., 2023 demonstrated that this resulted in better discriminatory power than 5% Blood Agar medium used in our study, which could have had a negative effect on our results for veterinary *P. aeruginosa* isolates. It may be that our FTIR validation process to optimise the culture scheme and COV determination, which constitutes the main and, perhaps, only complication of applying FTIR to routine laboratory settings, should be revisited and improved for typing veterinary *P. aeruginosa*. No correlation was found between FTIR spectroscopy and molecular methods in another study for *P. aeruginosa* strain classification (Oho et al., 2021). These authors analysed clinical *P. aeruginosa* from multiple institutions and concluded that differences in the measurement principles may account for classification mismatches of *P. aeruginosa* organisms; WGS data could help understanding the genomic variations between isolates that modify carbohydrate composition and therefore impact the FTIR spectrum. As this was a retrospective analysis of veterinary hospital isolates collected over several years, it is plausible that genomic changes which could be reflected in the carbohydrate composition of the bacterial cell wall may have occurred over time, and this may have affected *Pseudomonas aeruginosa* more than *Klebsiella pneumoniae* in light of its larger genome. The genetic background and population structure of *P. aeruginosa* isolates in the present study was greatly diversified; nonetheless a clear association was found between ST2644 isolates and the EH, particularly from the ICU (including colonisation, environmental and clinical isolates). This clone has been previously described in MDR virulent *P. aeruginosa* isolates from small animal hospitals in Japan (Hayashi et al., 2021) and it is herein reported in horses for the first time. Other potential high-risk clones identified in this study included ST395 and ST244, both associated to small animal clinical or environmental isolates. The former ST has been described among carbapenemase-negative carbapenem-nonsusceptible *P. aeruginosa* small animal clinical isolates in France (Haenni et al., 2017) whereas the latter is a global epidemic human clone often expressing acquired carbapenem-resistance genes and MDR/XDR profiles (Chen et al., 2014; del Barrio-Tofiño et al., 2020; Pérez-vázquez et al., 2020) that has also been reported in veterinary infections (Haenni et al., 2015) and hospital environments (Soonthornsit et al., 2023). Finally, *P. aeruginosa* isolated across multiple sink taps across the SAH1 belonged to ST446, a recently identified high-risk clone associated with multidrug resistance (Pincus et al., 2020).

The reasons behind numerous FTIR clusters containing isolates collected at broad time intervals (≥3 years), as herein seen for both organisms, warrants more focused prospective studies. For example, it would be useful to understand whether this is determined by the persistence of MDR-GNs in the hospital environments, their re-introduction or by the highly clonal nature of some isolates within certain bacterial species. Furthermore, larger prospective studies would be beneficial to understand and measure the impact of FTIR real-time hospital surveillance to infection prevention and control. Finally, it is important to bear in mind that, in our study, we compared the concordance of FTIR spectroscopy and WGS-based cluster analyses where only a subset of FTIR clustering isolates underwent the gold standard method (few isolates for each defined cluster, proportional to cluster size) which represents the main limitation of this work.

In conclusion, we found that FTIR spectroscopy accurately clustered veterinary *K. pneumoniae* isolates belonging to the same clone but may be less accurate in discriminating *P. aeruginosa* isolates. The resolution of this method was high for *K. pneumoniae* for both clinical/colonisation and environmental hospital isolates, suggesting that FTIR spectroscopy alone could provide sufficient information to support early and appropriate infection control measures for hospital outbreak and epidemiological surveillance of *K. pneumoniae.* The rapid turnaround time combined with its ease of performance, low cost per sample and lack of requirement for specialised training, bring considerable advantages over the current molecular reference methods (i.e., PFGE, MLST and WGS) for implementation into the routine workflow of a veterinary microbiology laboratory conducting infection control work. Here, we have shown that FTIR spectroscopy has the potential to become a valuable tool for rapid identification of *Klebsiella pneumoniae* (and potentially other pathogens) transmission events between patients and the veterinary clinical environment, therefore providing real-time surveillance information to aid infection prevention in veterinary settings.

## Data availability statement

The datasets presented in this study can be found in online repositories. The names of the repository/repositories and accession number(s) can be found at: ENA—PRJEB70897.

## Ethics statement

The animal studies were approved by University of Liverpool Veterinary Research Ethics Committee. The studies were conducted in accordance with the local legislation and institutional requirements. Written informed consent was obtained from the owners for the participation of their animals in this study.

## Author contributions

FZ: Data curation, Formal analysis, Investigation, Writing – original draft, Writing – review & editing. VS: Resources, Writing – review & editing. NM: Data curation, Formal analysis, Resources, Writing – review & editing. AL: Resources, Writing – review & editing. RJ: Resources, Writing – review & editing. CI: Resources, Writing – review & editing. GP: Funding acquisition, Writing – review & editing. SH: Data curation, Formal analysis, Resources, Writing – review & editing. DT: Conceptualization, Methodology, Project administration, Resources, Supervision, Writing – original draft, Writing – review & editing.

## Footnotes

^1^ http://rest.pubmlst.org/db/

^2^ https://github.com/jacarrico/goeBURST

^3^ http://www.comparingpartitions.info/
