# Corrigendum: Rapid typing of *Klebsiella pneumoniae* and *Pseudomonas aeruginosa* by Fourier-transform Infrared spectroscopy informs infection control in veterinary settings

**DOI:** 10.3389/fmicb.2024.1396367

**Published:** 2024-03-20

**Authors:** Flavia Zendri, Vanessa Schmidt, Norman Mauder, Anette Loeffler, Rosanne Ellen Jepson, Cajsa Isgren, Gina Pinchbeck, Sam Haldenby, Dorina Timofte

**Affiliations:** ^1^Department of Veterinary Anatomy, Physiology and Pathology, Institute of Infection, Veterinary and Ecological Sciences, University of Liverpool, Neston, United Kingdom; ^2^Department of Small Animal Clinical Science, Institute of Infection, Veterinary and Ecological Sciences, University of Liverpool, Neston, United Kingdom; ^3^Bruker Daltonics, Bremen, Germany; ^4^Department of Clinical Science and Services, Royal Veterinary College Hawkshead Campus, Hatfield, Hertfordshire, United Kingdom; ^5^Western Counties Equine Hospital Ltd., Culmstock, United Kingdom; ^6^Department of Livestock and One Health, Institute of Infection, Veterinary and Ecological Sciences, University of Liverpool, Neston, United Kingdom; ^7^Centre for Genomic Research, University of Liverpool, Liverpool, United Kingdom

**Keywords:** veterinary, infection control, Fourier-transform infrared (FTIR) spectroscopy, veterinary settings, companion animals, *Klebsiella pneumoniae*, *Pseudomonas aeruginosa*

In the published article, there was an error in affiliation 3. Instead of “School of Veterinary Science Small Animal Teaching Hospital, University of Liverpool, Neston, United Kingdom”, it should be “Bruker Daltonics, Bremen, Germany”.

The authors apologize for this error and state that this does not change the scientific conclusions of the article in any way. The original article has been updated.

